# Focused Chest Pain Assessment for Early Detection of Acute Coronary Syndrome: Development of a Cardiovascular Digital Health Intervention

**DOI:** 10.5334/gh.1194

**Published:** 2023-04-20

**Authors:** Mifetika Lukitasari, Sony Apriliyawan, Halidah Manistamara, Yurike Olivia Sella, Mohammad Saifur Rohman, Jitendra Jonnagaddala

**Affiliations:** 1 School of Population Health, UNSW Sydney, Kensington, Australia; 2 School of Nursing, Faculty of Health Sciences, Brawijaya University, Malang, East Java, Indonesia; 3 Cardiovascular Research Group, Faculty of Medicine, Brawijaya University, Malang, East Java, Indonesia; 4 Department of Cardiology and Vascular Medicine, Brawijaya University-Saiful Anwar General Hospital, Malang, East Java, Indonesia

**Keywords:** acute coronary syndrome, chest pain assessment, digital health

## Abstract

**Background:**

Chest pain misinterpretation is the leading cause of pre-hospital delay in acute coronary syndrome (ACS). This study aims to identify and differentiate the chest pain characteristics associated with ACS.

**Methods:**

A total of 164 patients with a primary complaint of chest pain in the ER were included in the study. ACS diagnosis was made by a cardiologist based on the WHO criteria, and the patients were interviewed 48 hours after their admission. Furthermore, every question was analysed using the crosstabs method to obtain the odds ratio, and logistic regression analysis was applied to identify the model of focused questions on chest pain assessment.

**Results:**

Among the samples, 50% of them had an ACS. Four questions fitted the final model of ACS chest pain focused questions: 1) Did the chest pain occur at the left/middle chest? 2) Did the chest pain radiate to the back? 3) Was the chest pain provoked by activity and relieved by rest? 4) Was the chest pain provoked by food ingestion, positional changes, or breathing? This model has 92.7% sensitivity, 84.1% specificity, 85% positive predictive value (PPV), 86% negative predictive value (NPV), and 86% accuracy. After adjusting for gender and diabetes mellitus (DM), the final model has a significant increase in Nagelkerke R-square to 0.737 and Hosmer and Lemeshow test statistic of 0.639.

**Conclusion:**

Focused questions on 1) left/middle chest pain, 2) retrosternal chest pain, 3) exertional chest pain that is relieved by rest, and 4) chest pain from food ingestion, positional changes, or breathing triggering can be used to rule out ACS with high predictive value. The findings from this study can be used in health promotion materials and campaigns to improve public awareness regarding ACS symptoms. Additionally, digital health interventions to triage patients’ suffering with chest pain can also be developed.

## 1. Introduction

Acute coronary syndrome (ACS), a cardiac emergency, requires timely reperfusion to improve patients’ clinical outcomes. A previous study among 5,243 STEMI patients treated by primary coronary intervention suggested that every reduction of door to balloon time by 30 minutes resulted in continuous one-year mortality reduction [1]. In addition, previous meta-analysis suggested that early invasive strategy for NSTEMI patients was associated with significant reduction in both composite end point and all-cause mortality [2]. Therefore, it is imperative to reduce the total ischemic time.

Pre-hospital delay, a major contributor of prolonged total ischemic time, is mainly caused by patients’ decision delay. Decision time constituted 60% of the total pre-hospital delay period, while home-to-hospital delay accounted for 40% [3]. Chest pain symptom misinterpretation at the onset of ACS has been confirmed as a significant reason of patients’ decision delay [4,5,6]. A previous study suggested that many adults in the United States remain unaware of the symptoms of and appropriate response to a myocardial infarction [7]. Despite several awareness campaigns, women’s decision delay has persisted over time due to lack of ACS awareness [8,9]. Therefore, early identification of chest pain suggestive of ACS is imperative to reduce the pre-hospital delay. A considerable number of chest pain patient come to primary care that suggested requirement of chest pain assessment to differentiate ACS from non-ACS patients. In fact, more than 50–75% of the seven million patients with chest pain are admitted to the hospital because the initial clinical evaluation is not sufficient to rule in or rule out ACS [10]. Hence, it is important to emphasize the chest pain assessment and to discriminate chest pain of ACS from non-cardiac chest pain.

Digital health has grown rapidly in various aspects of cardiovascular health, such as primary, secondary, and tertiary prevention of ACS. A systematic review and meta-analysis demonstrated that digital health intervention for cardiovascular disease (CVD) prevention significantly improved CVD outcomes and showed a positive impact on CVD risk factors [11,12]. Most digital health interventions are focused on public’s health literacy improvement and risk factor management in CVD prevention [11,12,13]. Considering the improvement of decision delay reduction, digital health should address the issue of ACS symptoms awareness and early identification. However, to our knowledge, there is limited number of digital health intervention in CVD that address patients’ awareness and early identification to ACS symptoms.

With an exponential growth of machine learning algorithm, the significance of artificial intelligence as a component of CVD diagnostic process has been steadily improving diagnostic accuracy and efficiency [14]. A previous study regarding machine learning in predicting the likelihood of acute myocardial infarction suggested that myocardial ischemic injury index incorporating age, sex, and paired high sensitivity cardiac troponin I concentrations showed a better performance compared to European Society of Cardiology 0/3-hour pathway (sensitivity, 82.5% [74.5–88.8%]; specificity, 92.2% [90.7–93.5%]) [15]. Moreover, pre-hospital electrocardiography-based machine learning was developed to predict acute coronary syndrome [10]. In addition, Noh et al. developed a machine learning-based approach for ACS requiring revascularization prediction [16]. However, to our knowledge only one study predicted acute myocardial infarction based on its sign and symptoms [17].

Identification of chest pain characteristics that may rule out ACS is imperative to facilitate patients’ self-identification for ACS awareness while suffering chest pain. Previous studies that used prediction models to assess symptom magnitude in ACS diagnosis showed varied results [18,19,20,21,22,23]. They showed that chest pain characteristics such as retrosternal pain, shoulder pain, arm pain, sweating, nausea, and vomiting were predictors of ACS; however, these studies suggested distinct chest pain characteristics from the recent guideline by American Heart Association [24]. Moreover, some of those studies included the presence of ACS’s risk factors in the questionnaire of ACS. In fact, most patients are unaware of their own ACS risk factors while suffering from chest pain [25,26,27]. In addition, previous studies reported that women with ACS experience different symptoms compared to that of men with ACS [18,19,22,28,29,30]. Moreover, a study by Banco et al. suggested that young women (aged ≤55 years) presenting with chest pain at the emergency department (ED) were less likely to be admitted to the hospital or to observation compared with young men [31]. Chest pain characteristics should be elucidated to obtain high sensitivity and specificity predictors of ACS to facilitate patients’ awareness of ACS.

With the main intention of developing a digital acute coronary syndrome early identification program, the primary objective of this study is to develop a model that characterizes patients’ chest pain characteristics which can be used to rule out ACS with high predictive value. The second objective of this study is to differentiate the ACS suggestive chest pain characteristics between men and women. High predictive value chest pain characteristics obtained from this study led to design and development of a cardiovascular digital health application called DETAK C. Digital health interventions like DETAK C can facilitate public awareness and patients’ decision improvement to ACS suggestive chest pain through a digital health.

## 2. Materials and Methods

### 2.1. Study design and setting

**Figure 1:**
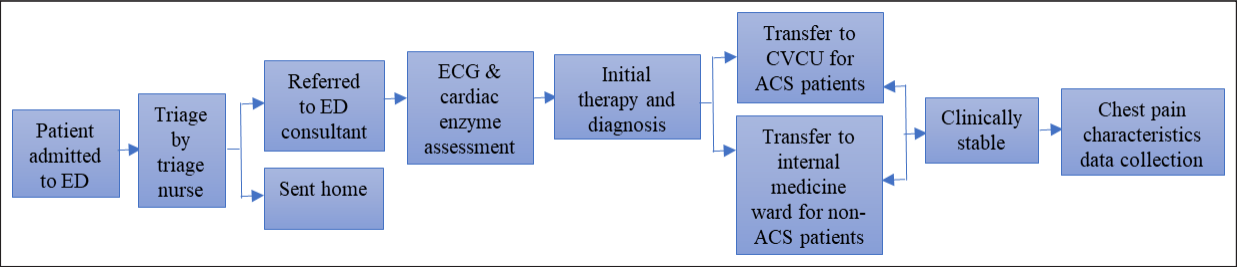
**Clinical workflow of patients.** ED, Emergency department; ECG, Electrocardiography; CVCU, Cardiovascular care unit.

A retrospective study was conducted at Saiful Anwar General Hospital, and data were collected by three trained nurse students from similar academic year using a paper-based chest pain assessment questionnaire. It was developed by the National Institute for Health and Care Excellence [32]. The study was approved by the Institutional Ethics Committee of Saiful Anwar General Hospital, with the approval number 400/016/K.3/302/2019. The workflow of this study is depicted in Figure 1.

### 2.2. Selection of subjects

**Figure 2:**
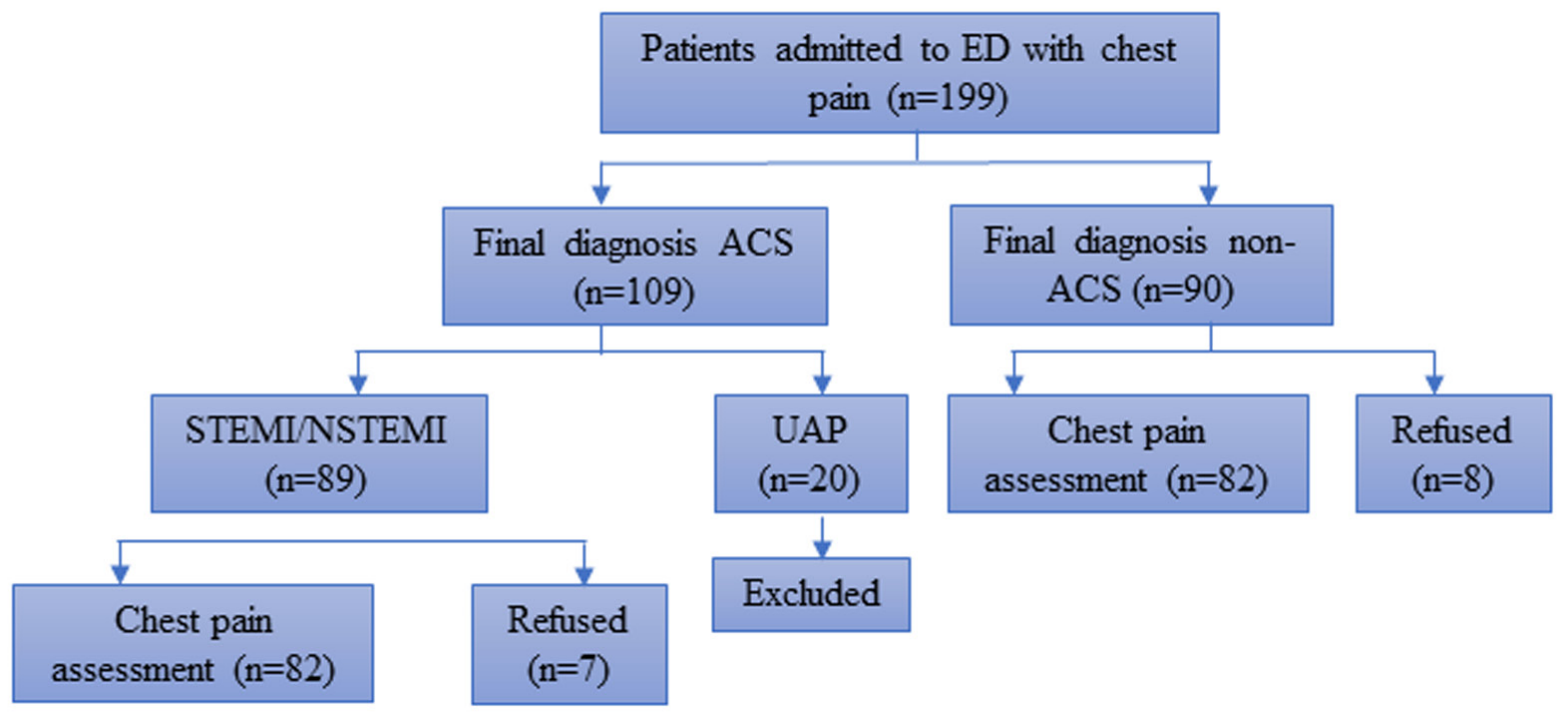
**Recruitment of study participants.** ED, Emergency department; ACS, Acute coronary syndrome; STEMI, ST elevation myocardial infarction; NSTEMI, non-ST elevation myocardial infarction.

A total of 164 patients with a primary complaint of chest pain were consecutively selected between January and February 2019. The sample was calculated by the formula of sample size for case control study with 1:1 ratio and the minimum sample size was 56 patients each group. The diagnosis of ACS (ST-elevation myocardial infarction [STEMI] and non ST-elevation myocardial infarction [NSTEMI]) was made by a cardiologist based on the following criteria: STEMI was defined by the onset of a persistent ST-elevation on ECG, considered suggestive in the following cases: 1) at least two continuous leads with ST-segment elevation > 0.2 mV in leads V1-V3 or > 0.1 mV in leads V4-V9, V3R, and V4R, or 2) left bundle branch block with the presence of concordant ST-segment elevation. In addition, non-STEMI was diagnosed based on compatible clinical presentation and ECG abnormalities in two continuous leads, ST-segment depression or T-wave changes, and elevated cardiac troponin levels higher than the 99^th^ percentile. Non-ACS patients were those who did not meet the ACS diagnosis criteria. The patients were interviewed by trained nursing students 48 hours after their admission or after transferred from cardiovascular care unit. The selection of subjects is depicted in Figure 2.

### 2.3. Analysis

All questions were analysed using the crosstabs method to measure odds ratios. Logistic regression analysis by SPSS software with α = 0.05 was used to identify odd ratio of each question and to generate the best model for chest pain assessment. The ROC curve was generated from the predictive value of the questions to obtain the sensitivity and specificity of the models.

## 3. Results

### 3.1 Baseline characteristics

This study included 164 patients, comprising 82 patients with ACS and 82 patients without ACS. The baseline characteristics of the patients are presented in Table 1, which contains the demographic data and clinical characteristics. The proportion of classic risk factors for ACS, such as older age, type 2 diabetes mellitus (type 2 DM), hypertension, dyslipidaemia, and family history of cardiovascular events, was significantly higher in the ACS group than in the non-ACS group (p < 0.05).

**Table 1:** Baseline characteristics.


		DIAGNOSIS		P-VALUE

		ACS (N = 82)	NON-ACS (N = 82)	

Sex				

	Male	63 (76.8%)	49 (59.8%)	0.019

	Female	19 (23.2%)	33 (40.2%)	

Age (years)				

	Male <55	16 (19.5%)	22 (26.8%)	≤0.005

	Male ≥55	47 (57.3%)	28 (34.1%)	

	Female <60	9 (11%)	24 (29.3%)	

	Female ≥60	10 (12.2%)	8 (9.8%)	

Occupation				

	Public servant	8 (9.8%)	10 (12.2%)	0.092

	Private servant	48 (58.5%)	58 (70.7%)	

	Unemployed	26 (31.7%)	14 (17.1%)	

Education				

	Not completed elementary school	2 (2.4%)	0 (0.0%)	0.061

	Elementary school	20 (24.4%)	17 (20.7%)	

	Junior high School	21 (25.6%)	34 (41.5%)	

	Senior high school	31 (37.8%)	19 (23.2%)	

	Bachelor’s degree	8 (9.8%)	12 (14.6%)	

Risk Factors				

Diabetes mellitus				

	Yes	35 (42.7%)	9 (11.0%)	≤0.000

	No	47 (57.3%)	73 (89.0%)	

Smoking				

	Yes	52 (63.4%)	42 (51.2%)	0.114

	No	30 (36.6%)	40 (48.8%)	

Hypertension				

	Yes	52 (63.4%)	24 (29.3%)	≤0.000

	No	30 (36.6%)	58 (70.7%)	

Dyslipidaemia				

	Yes	12 (14.6%)	4 (4.9%)	0.035

	No	70 (85.4%)	78 (95.1%)	

Family history of cardiovascular event				

	Yes	8 (9.8%)	0 (0.0%)	≤0.004

	No	74 (90.2%)	82 (100.0%)	

History of a heart attack				

	Yes	20 (24.4%)	2 (2.4%)	≤0.01

	No	62 (75.6%)	80 (97.6%)	


ACS, acute coronary syndrome.

### 3.2 Chest pain characteristics of patients with ACS and non-ACS

The chest pain characteristics of patients with and without ACS are presented in Table 2. Furthermore, left/middle chest pain which radiated to the back was the first experience of chest pain, and chest pain that appeared at rest was more significant in patients with ACS as compared to that in patients without ACS. The ACS group also had pain that persisted for a longer duration and was not provoked by food ingestion, positional changes, or breathing compared to that of non-ACS patients.

**Table 2:** Odds ratio for each question in the chest pain assessment.


QUESTIONS		DIAGNOSIS		P-VALUE	OR (95% CI)

		ACS (N = 82)	NON-ACS (N = 82)		

Was the chest pain located at the left/middle chest?					

	Yes	72 (87.8%)	58 (70.7%)	0.012	2.979 (1.31–6.72)

	No	10 (12.2%)	24 (29.3%)		

Did the chest pain radiate to the neck?					

	Yes	9 (11.0%)	7 (8.5%)	0.792	1.321 (0.46–3.73)

	No	73 (89.0%)	75 (91.5%)		

Did the chest pain radiate to the back?					

	Yes	47 (57.3%)	14 (17.1%)	0.000	6.522 (3.16–13.43)

	No	35 (42.7%)	68 (82.9%)		

Did the chest pain radiate to the jaw?					

	Yes	3 (3.7%)	3 (3.7%)	1.000	1.000 (0.19–5.10)

	No	79 (96.3%)	79 (96.3%)		

Did the chest pain radiate to the left arm?					

	Yes	10 (12.2%)	7 (8.5%)	0.608	1.488 (0.53–4.12)

	No	72 (87.8%)	75 (91.5%)		

Did the chest pain radiate to the epigastric?					

	Yes	15 (18.3%)	13 (15.9%)	0.836	1.188 (0.52–2.68)

	No	67 (81.7%)	69 (84.1%)		

Was this the first chest pain experience?					

	Yes	62 (75.6%)	80 (97.6%)	0.000	0.078 (0.01–0.34)

	No	20 (24.4%)	2 (2.4%)		

Was the chest pain provoked by activity and relieved by rest?					

	Yes	13 (15.9%)	71 (86.6%)	0.000	34.25 (14.37–81.66)

	No	69 (84.1%)	11 (13.4%)		

Did the chest pain appear during mild activity?					

	Yes	15 (18.3%)	11 (13.4%)	0.521	1.445 (0.62–3.36)

	No	67 (81.7%)	71 (86.6%)		

Did the chest pain appear at rest?					

	Yes	32 (39.0%)	0 (0.0%)	0.000	N/A

	No	50 (61.0%)	82 (100%)		

Did you have any previous episode of chest pain?					

	Yes	20 (24.4%)	7 (8.5%)	0.006	3.456 (1.37–8.70)

	No	62 (75.6%)	75 (91.5%)		

Compared to the previous chest pain episode, was this episode provoked by any activities that were less intense than in the previous episode? (n = 27)					

Yes		11 (55%)	0 (0.0%)	0.036	N/A

No		9 (45%)	7 (100.0%)		

Was the duration of chest pain more than 20 minutes?					

Yes		82 (100.0%)	19 (23.2%)	0.000	N/A

No		0 (0.0%)	63 (76.8%)		

Did the chest pain result in a pressured/crushing sensation?					

Yes		36 (43.9%)	30 (36.6%)	0.426	1.357 (0.72–2.57)

No		46 (56.1%)	52 (63.4%)		

Was the chest pain burning or stabbing?					

Yes		41 (50.0%)	59 (72.0%)	0.007	2.565 (1.34–4.90)

No		41 (50.0%)	23 (28.0%)		

Was the chest pain provoked by food ingestion or positional changes or breathing?					

Yes		5 (6.1%)	29 (35.4%)	0.000	8.426 (3.06–23.17)

No		77 (93.9%)	53 (64.6%)		


ACS, acute coronary syndrome; OR, odds ratio; CI, confidence interval.

### 3.3 Odds ratio for each question in the chest pain assessment

The odds ratios for each question in the chest pain assessment are presented in Table 2. The results showed that the highest odds ratio were obtained for the following questions: had the chest pain located in the left/middle chest (OR = 2.979; 95% CI = 1.31–6.72), chest pain that radiated to the back (OR = 6.522; 95% CI = 3.16–13.43), first chest pain (OR = 0.078; 95% CI = 0.01–0.34), chest pain provoked by activity and relieved by rest (OR = 34.25; 95% CI = 14.37–81.66), an episode of chest pain (OR = 3.456; 95% CI = 1.37–8.70), burning or stabbing chest pain (OR = 2.565; 95% CI = 1.34–4.90), and the chest pain was provoked by food ingestion, positional changes, or breathing (OR = 8.426; 95% CI = 3.06–23.17).

### 3.4 Multivariate analysis of chest pain assessment

Multivariate logistic regression was applied to determine model for focused questions in chest pain assessment, and those with p-values < 0.05 were included in the analysis. The focused questions on ACS prediction after multivariate logistic regression analysis are presented in Table 3. Model of focused questions in chest pain assessment consists of the following questions: 1) was the chest pain located at the left/middle chest? 2) did the chest pain radiate to the back? 3) was the chest pain provoked by activity and relieved by rest? 4) was the chest pain provoked by food ingestion, positional changes, or breathing? This model has Nagelkerke R-square of 0.685, Hosmer and Lemeshow test statistic of 0.422, and Omnibus test statistic of 0.000. Furthermore, this model was adjusted for gender and type 2 diabetes mellitus risk factor as presented in Table 4. After the adjustment, the Nagelkerke R-square as well as the Hosmer and Lemeshow of the final model increased to 0.737 and 0.639 respectively. In the final model, the OR of the following questions significantly increased: 1) did the chest pain radiate to the back? (yes) from OR = 10.012; 95% CI = 3.254–30.810 to OR = 12.384; 95% CI = 3.378–45.405; 2) was the chest pain provoked by activity and relieved by rest? (no) from OR = 27.546; 95% CI = 10.029–75.658 to OR = 34.543; 95% CI = 10.761–110.853, while the remaining questions suggested a slight decrease in their OR.

**Table 3:** Multivariate analysis of chest pain assessment.


VARIABLES	P-VALUE	EXP (β)	95% CI	

			LOWER	UPPER

**Step 1**				

Was the chest pain located at the left/middle chest? (yes)	0.015	5.265	1.380	20.081

Did the chest pain radiate to the back? (yes)	0.000	9.108	2.828	29.336

Was this the first chest pain experience? (yes)	0.032	26.300	1.322	523.039

Was the chest pain provoked by activity and relieved by rest? (no)	0.000	24.599	8.625	70.163

Did you have any previous chest pain? (yes)	0.121	0.143	0.012	1.677

Was the chest pain burning or stabbing? (yes)	0.871	0.913	0.306	2.728

Was the chest pain provoked by food ingestion, positional changes, or breathing? (no)	0.035	5.524	1.126	27.106

**Step 2**				

Was the chest pain located at the left/middle chest? (yes)	0.015	5.184	1.380	19.473

Did the chest pain radiate to the back? (yes)	0.000	9.020	2.822	28.834

Was this the first chest pain experience? (yes)	0.032	25.402	1.319	489.306

Was the chest pain provoked by activity and relieved by rest? (no)	0.000	24.452	8.605	69.481

Did you have any previous chest pain? (yes)	0.124	0.144	0.012	1.697

Was the chest pain provoked by food ingestion, positional changes, or breathing? (no)	0.029	5.284	1.185	23.572

**Step 3**				

Was the chest pain located at the left/middle chest? (yes)	0.020	4.639	1.267	16.982

Did the chest pain radiate to the back? (yes)	0.000	8.737	2.824	27.026

Was this the first chest pain experience? (yes)	0.114	4.209	0.709	24.965

Was the chest pain provoked by activity and relieved by rest? (no)	0.000	23.604	8.485	65.663

Was the chest pain provoked by food ingestion or positional changes or breathing? (no)	0.041	4.711	1.063	20.876

**Step 4**				

Was the chest pain located at the left/middle chest? (yes)	0.021	4.461	1.257	15.828

Did the chest pain radiate to the back? (yes)	0.000	10.012	3.254	30.810

Was the chest pain provoked by activity and relieved by rest? (no)	0.000	27.546	10.029	75.658

Was the chest pain provoked by food ingestion, positional changes, or breathing? (no)	0.031	4.788	1.152	19.908


CI, confidence interval.

**Table 4:** Multivariate analysis of chest pain assessment adjusted for gender and type 2 diabetes mellitus risk factor.


VARIABLES	P VALUE	EXP (β)	95% CI	

			LOWER	UPPER

Gender (Male)	0.061	2.986	0.952	9.369

Diabetes Mellitus (yes)	0.005	6.393	1.777	23.004

Was the chest pain located at the left/middle chest? (yes)	0.000	4.381	1.047	18.333

Did the chest pain radiate to the back? (yes)	0.052	12.384	3.378	45.405

Was the chest pain provoked by activity and relieved by rest? (no)	0.000	34.543	10.761	110.853

Was the chest pain provoked by food ingestion, positional changes, or breathing? (no)	0.043	4.679	0.988	22.158


CI, confidence interval.

### 3.5 Chest pain characteristics difference between men and women

More similarities than differences were observed in ACS suggestive chest pain characteristics between men and women except for the chest pain quality as shown in Table 5. Retrosternal radiation of chest pain was more common in women than in men with ACS (women 55.6% vs. men 63.2%; gender interaction p = 0.247). Burning or stabbing chest pain was more common in men than in women with ACS (women 31.6% vs. men 55.6%; gender interaction p = 0.012). Exertional chest pain that is not relieved by rest was reported by comparable proportion of men and women with ACS (women 89.5% vs. men 82.5%; gender interaction p = 0.428). The absence of positional, breathing, and food ingestion related chest pain was reported by 98.4% of men versus 78.9% of women with ACS; gender interaction p = 0.082.

**Table 5:** Subgroup Analysis Chest Pain Characteristic Difference between Men and Women.


QUESTIONS	MEN (N = 112)				WOMEN (N = 52)				P-VALUE INTERACTION
	
	ACS (N = 63)	NON-ACS (N = 49)	P VALUE	OR	ACS (N = 19)	NON-ACS (N = 33)	P VALUE	OR	

Was the chest pain located at the left/middle chest?									

yes	55 (87.3)	36 (73.5)	0.063	2.483 (0.936–6.587)	17 (89.5)	22 (66.7)	0.135	4.250 (0.829–21.782)	0.360
	
no	8 (12.7)	13 (26.5)			2 (10.5)	11 (33.3)			

Did the chest pain radiate to the neck?									

yes	5 (7.9)	4 (8.2)	1.000	0.970 (0.246–3.821)	4 (21.1)	3 (9.1)	0.400	2.667 (0.528–13.477)	0.277
	
no	58 (92.1)	45 (91.8)			15 (78.9)	30 (90.9)			

Did the chest pain radiate to the back?									

yes	35 (55.6)	10 (20.4)	0.000	4.875 (2.075–11.453)	12 (63.2)	4 (12.1)	0.000	12.429 (3.063–50.431)	0.247
	
no	28 (44.4)	39 (79.6)			7 (36.8)	29 (87.9)			

Did the chest pain radiate to the jaw?									

yes	2 (3.2)	0 (0)	0.503	N/A	1 (5.3)	3 (9.1)	1.000	0.556 (0.054–5.752)	0.062
	
no	61 (96.8)	49 (100)			18 (94.7)	30 (90.9)			

Did the chest pain radiate to the left arm?									

yes	9 (14.3)	3 (6.1)	0.224	2.556 (0.653–10.002)	1 (5.3)	4 (12.1)	0.641	0.403 (0.042–3.894)	0.830
	
no	54 (85.7)	46 (93.9)			18 (94.7)	29 (87.9)			

Did the chest pain radiate to the epigastric?									

yes	11 (17.5)	7 (14.3)	0.650	2.556 (0.653–10.002)	4 (21.1)	6 (18.2)	1.000	1.200 (0.292–4.934)	0.618
	
no	52 (82.5)	42 (85.7)			15 (78.9)	27 (81.8)			

Was this the first chest pain experience?									

yes	16 (25.4)	2 (4.1)	0.003	1.269 (0.453–3.559)	15 (78.9)	0 (0)	0.014	N/A	0.144
	
no	47 (74.6)	42 (95.9)			4 (21.1)	33 (100)			

Did the chest pain appear during mild activity?									

yes	11 (17.5)	5 (10.2)	0.276	1.862 (0.601–5.767)	4 (21.1)	6 (18.2)	1.000	N/A	0.421
	
no	52 (82.5)	44 (89.8)			15 (78.9)	27 (81.8)			

Did the chest pain provoked by activity and relieved by rest?									

yes	11 (17.5)	44 (89.8)	0.000	41.600 (13.428–128.882)	2 (10.5)	27 (81.8)	0.000	38.250 (6.908–211.799)	0.428
	
no	52 (82.5)	5 (10.2)			17 (89.5)	6 (18.2)			

Did the chest pain appear at rest?									

yes	23 (36.5)	0 (0)	0.106	N/A	9 (47.4)	0 (0)	0.000	N/A	0.628
	
no	40 (63.5)	49 (100)			10 (52.6)	33 (100)			

Did you have any previous episode of chest pain?									

yes	15 (23.8)	5 (10.2)	0.062	2.750 (0.923–8.193)	5 (26.3)	2 (6.1)	0.085	5.536 (0.955–32.082)	0.481
	
no	48 (76.2)	44 (89.8)			14 (73.7)	31 (93.9)			

Compared to the previous chest pain episode, was this episode provoked by any activities that were less intense than in the previous episode? (n = 20)									

yes	7 (46.7)	0 (0)	0.114	N/A	4 (80)	0 (0)	0.143	N/A	0.314
	
no	8 (53.3)	5 (100)			1 (20)	2 (100)			

Was this chest pain episode provoked by daily activities as in the case of previous chest pain? (n = 20)									

yes	8 (53.3)	5 (100)	0.114	N/A	1 (20)	2 (100)	0.143	N/A	0.314
	
no	7 (46.7)	0 (0)			4 (80)	0 (0)			

Was the duration of chest pain more than 20 minutes?									

yes	63 (100)	13 (26.5)	0.000	N/A	19 (100)	6 (18.2)	0.000	N/A	0.016
	
no	0 (0)	36 (73.5)			0 (0)	27 (81.8)			

Did the chest pain result in a pressured/crushing sensation?									

yes	27 (42.9)	15 (30.6)	0.258	1.700 (0.774–3.731)	9 (47.4)	15 (45.5)	0.894	1.080 (0.348–3.349)	0.294
	
no	36 (57.1)	34 (69.4)			10 (52.6)	18 (54.5)			

Was the chest pain burning or stabbing?									

yes	35 (55.6)	16 (32.7)	0.026	2.578 (1.186–5.606)	6 (31.6)	7 (21.2)	0.406	1.714 (0.478–6.151)	0.012
	
no	28 (44.4)	33 (67.3)			13 (68.6)	26 (78.8)			

Was the chest pain provoked by food ingestion or positional changes or breathing?									

yes	1 (1.6)	18 (36.7)	0.000	36.000 (4.592–282.260)	4 (21.1)	11 (33.3)	0.526	1.875 (0.501–7.013)	0.082
	
no	63 (98.4)	31 (63.3)			15 (78.9)	22 (66.7)			


ACS, Acute Coronary Syndrome.

### 3.6 Sensitivity and specificity of chest pain assessment

**Figure 3:**
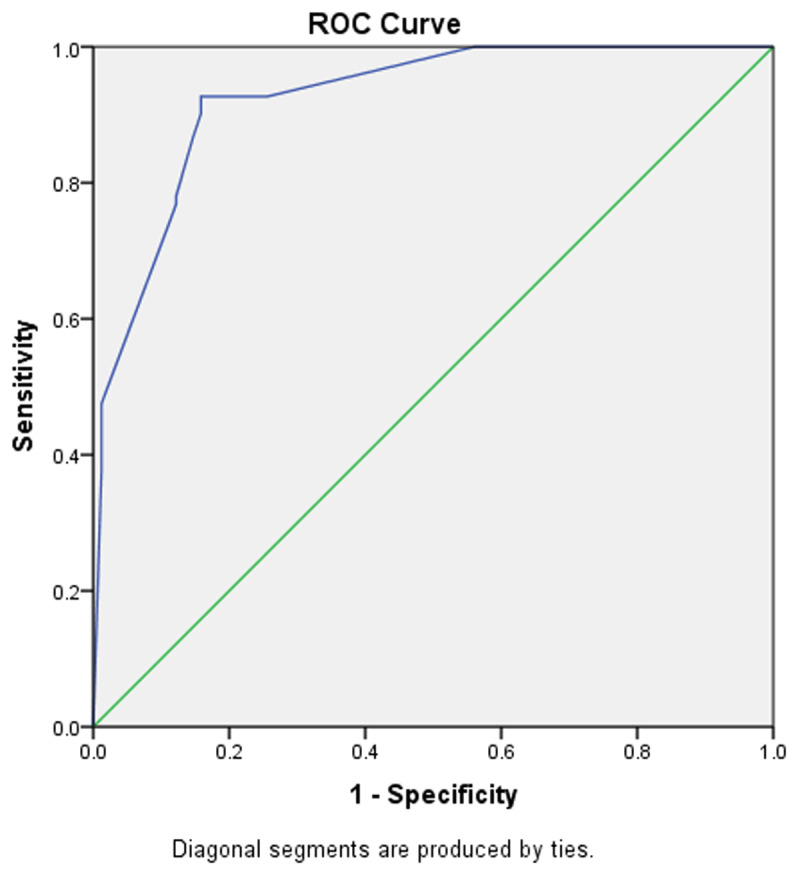
**Receiver operating characteristic curve for chest pain assessment.** With a sensitivity of 92.1% and 1-specificity of 84.1%, a cut-off point of 0.37 was obtained. ROC, receiver operating characteristic; AUC, area under the curve.

Using receiver operating characteristic (ROC) curve analysis, the cut-off point for chest pain assessment was calculated. ROC analysis of chest pain assessment showed that the area under the curve was 0.925 (95% CI = 0.885–0.964) with a p-value < 0.001. The ROC curve is depicted in Figure 3. The cut-off point was 0.37 with 92.7% sensitivity, 84.1% specificity, 85% PPV, 86% NPV, and 86% accuracy.

## 4. Discussion

In our study, predictive variables of ACS in both men and women were consistent with the typical chest pain characteristics. More similarities than differences observed in chest pain characteristics associated with the diagnosis of ACS for women and men. The presence of centrally located retrosternal, and exertional chest pain were associated with ACS in both sexes. Moreover, positional, breathing, and food ingestion related chest pain was associated with non-ACS diagnosis. The cut-off values of sensitivity, specificity, PPV, and NPV indicated four questions as focused assessments for the early detection of ACS. The questions were: 1) Did the chest pain occur at the left/middle chest? 2) Did the chest pain radiate to the back? 3) Was the chest pain provoked by activity and relieved by rest? 4) Was the chest pain provoked by food ingestion, positional changes, or breathing? These questions were more effective in assisting ACS detection and the multivariate analysis showed a Nagelkerke R-square value of 0.685, which explained 68% of ACS detection. After adjusting for gender and type 2 diabetes mellitus risk factor the Nagelkerke R-square value significantly increased to 0.737, which explained 74% of ACS detection.

Chest pain located in the left or middle of the chest suggested ACS with an adjusted OR of 4.461 (95% CI = 1.257–15.828) and provides an important clue for ACS detection. This centrally located chest pain is due to cardiac ischaemia, unlike the peripherally located chest pain, and previous studies have confirmed comparable results to those of our study [33,34,35]. In contrast, Bosner et al. reported that pain location of ACS patients was similar to that of non-ACS patients [36]. Moreover, our study revealed that there were no differences in the presence of left/central chest pain between the sexes or age groups. This finding was comparable to the previous study that demonstrated the similarity of chest pain location between women and men with coronary heart disease [36]. Conversely, previous study revealed that men ruled-in for ACS were more likely to experience mid-chest pain compared to women [30]. But in our study, the analyses were not adjusted for sex, age, and history of diabetes mellitus, and a small sample size might be a bias in this study.

The presence of chest pain radiation also suggests ACS. This is due to the left position of the heart and radiating pain along the left cervical nerve roots. Our results indicated that pain radiation to the back is suggestive of ACS with an OR of 10.012 (95% CI = 3.254–30.810). Our study demonstrated that retrosternal pain was experienced by both men and women with ACS. In contrast, previous study showed that chest pain radiation to the back was significantly reported more frequent in women than in men [32,37]. Moreover, previous study reported pain radiating toward the left shoulder, arm, jaw, and neck accounted for approximately 22.7% of ACS patients [34]. Mirzaei et al. reported that a higher frequency of pain radiation to the jaw/neck/throat was observed in females ruled-in for ACS, though, the difference in pain radiation to the jaw/neck/throat did not differ between ACS and non-ACS patients [30].

The quality of chest pain in patients with ACS is usually reported as dull, burning, throbbing, or being pressured, and is generally considered to be cardiac ischaemia. A previous study showed that the burning quality had an odds ratio of 3.0 (95% CI = 1.1–8.4) in myocardial infarction diagnosis [38]. Sharp chest pain showed sensitivity and specificity of 8%–16% and 59%–70% respectively, in myocardial infarction diagnosis with a likelihood ratio of 0.3 (95% CI = 0.2–0.5) [39]. Our study indicated that sharp and stabbing chest pain was associated with ACS diagnosis, however, multivariate analysis revealed that it was not sensitive and specific to ACS. This result contradicted with a previous study, which showed that sharp and stabbing chest pain more powerfully differentiated non-ischaemic from ischaemic pain. Furthermore, previous study suggested that pain described as sharp or stabbing significantly decreased the likelihood of chest pain representing AMI [40,41]. A recent study indicated that both men and women reported similar chest pain quality as pressured, like heavy or tightening [18]; in contrast our study showed that pressured/crushing chest pain sensation was not the predictor of ACS.

Precipitating and aggravating factors of chest pain are predictors of ACS. Chest pain precipitation, such as body positional change, breathing, and eating, indicated non-ACS chest pain. However, there was no significant association between heavy exertion, resting chest pain, and ACS diagnosis. Conversely, exercise was significantly associated with angina [42,43,44]. However, the relationship between exercise and AMI remains unclear. Ayerbe et al. stated that heavy exertion within the hour before the event was common in patients with myocardial infarction [45]. Therefore, a correlation between exercise and AMI was confirmed. When exertional pain is lacking, the likelihood of AMI decreases, and chest pain relieved by rest indicates non-ACS. Rest characteristically relieves the pain associated with stable angina within 1 to 5 minutes [46]. When pain persists for longer than 10 minutes after rest, the patient is traditionally considered to experience unstable angina, AMI, or non-cardiac pain. This lack of significance makes it unclear even when rest is helpful in differentiating ACS from non-cardiac pathology [47].

Timely identification and diagnosis of ACS could be challenging in clinical practice and community. Patients’ prehospital delay can be due to lack of knowledge about the ACS symptoms, inability to interpret ACS symptoms, or confusion between ACS and other symptoms related to the upper respiratory or gastrointestinal tracts [48]. Moreover, women with ACS have longer patient delays, prehospital healthcare delays, hospital delays, and total healthcare delays [49]. Previous study also suggested that young women presenting to the ED with chest pain were less likely to be admitted to the hospital for evaluation compared to that of their counterparts and they also waited longer to be evaluated by physician thus, it is essential to conduct further study investigating chest pain characteristics suggesting ACS in young women [31]. This study will be beneficial for either primary care or ED while examining young women with ACS to prevent further delays.

**Figure 4:**
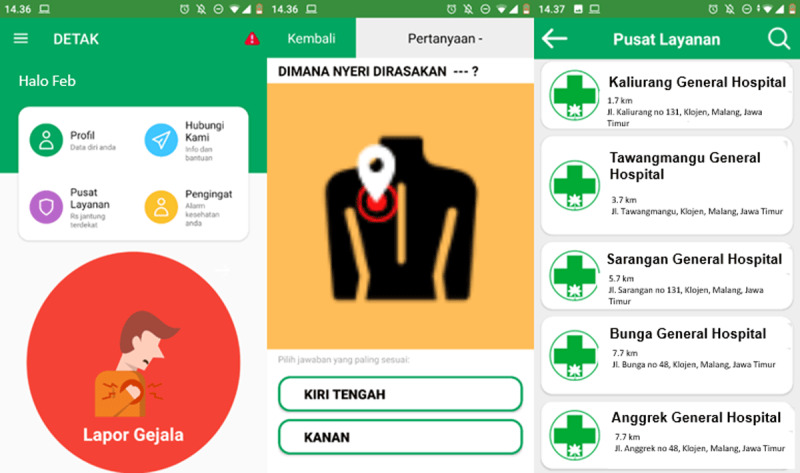
**DETAK C mobile application user interface.** DETAK C mobile application collecting chest pain characteristics information from the patient, analysing the data, and guiding the patient to the appropriate hospital.

Machine learning techniques, one of artificial intelligence application in cardiovascular medicine, has many possible advantages and prospects. Many studies have demonstrated machine learning power for CVD diagnosis and predictive analytics for personalized therapies [50]. A meta-analysis suggested that machine learning may be useful as an initial triage tool for ruling out ACS with pooled sensitivity and specificity were 0.95 and 0.90, respectively. The positive predictive values ranged from 0.64 to 1.0, and the negative predictive values ranged from 0.91 to 1.0 [51]. As decision delay contributed significantly to treatment delay the development of artificial intelligence assisting chest pain patient decision to appropriate hospital is essentially required. The results of this study formed basis for the development of mobile application namely DETAK C that already uploaded to Google Play Store (Figure 4). We developed a mobile application to triage patient with chest pain to appropriate care provider. As such patients suffering ACS suggestive chest pain may decide to visit primary percutaneous intervention capable hospital as soon as possible. The overall objective of this digital health intervention is to shorten the pre-hospital delay. This will be investigated in our future work. Moreover, the features of DETAK C will be improved by the addition of preventive and rehabilitative features to improve high-risk population awareness on ACS. The contribution of DETAK C mobile application in ACS prevention and rehabilitation will be investigated through a randomized controlled trial.

In this study, the samples were collected from cardiovascular care unit wherein the availability of comorbidity data was limited. These facts may limit the generalization of this study. Moreover, the small size of this study limits the generalization to wider population settings.

## 5. Conclusions

Our study reveals that the following questions: 1) Did the chest pain occur at the left/middle chest? (yes); 2) Did the chest pain radiate to the back? (yes); 3) Was the chest pain provoked by activity and relieved by rest? (no); and 4) Was the chest pain provoked by food ingestion, positional changes, or breathing? (no) can be used to rule out ACS with high predictive value. These chest pain assessment questions can be used in health promotion materials and campaigns to improve public awareness regarding ACS symptoms. Our findings can also be used to design various digital health interventions that focus on prevention and management of cardiovascular disease.

## Data Accessibility Statement

The data used to support the findings of this study are available from the corresponding author upon request.

## Additional File

The additional file for this article can be found as follows:

10.5334/gh.1194.s1 Appendix. Chest Pain Assessment Questionnaire.
