# Supplementary material for: Focused Chest Pain Assessment for Early Detection of Acute Coronary Syndrome: Development of a Cardiovascular Digital Health Intervention
Source: Glob Heart. 2023 Apr 20;17(1):18. doi: 10.5334/gh.1194 (PMC10120604; doi:10.5334/gh.1194)
Supplement: Appendix. — Chest Pain Assessment Questionnaire. [file gh-18-1-1194-s1.pdf]

Appendix - Chest Pain Assessment Questionnaire

Name	:
Address	:
Phone number	:
Gender	:
Age	:
Register number	:
Occupation	:
Education	:

No	Question	Answer	
1	What are the patients' risk factors?	☐ Smoking<br>☐ Dyslipidaemia<br>☐ Hypertension<br>☐ Diabetes Mellitus<br>☐ Chronic kidney disease<br>☐ History of heart attack<br>☐ Parents' history of heart attack	
2	Was the chest pain located at the left/middle chest?	☐ Yes	☐ No
3	Did the chest pain radiate to the neck?	☐ Yes	☐ No
4	Did the chest pain radiate to the back?	☐ Yes	☐ No
5	Did the chest pain radiate to the jaw?	☐ Yes	☐ No
6	Did the chest pain radiate to the left arm?	☐ Yes	☐ No

7	Did the chest pain radiate to the epigastric?	☐ Yes	☐ No
8	Was this the first chest pain experience?	☐ Yes	☐ No
9	Did the chest pain appear during mild activity?	☐ Yes	☐ No
10	Did the chest pain provoked by activity and relieved by rest?	☐ Yes	☐ No
11	Did the chest pain appear at rest?	☐ Yes	☐ No
12	Did you have any previous episode of chest pain?	☐ Yes	☐ No
13	Compared to the previous chest pain episode, was this episode provoked by any activities that were less intense than in the previous episode?	☐ Yes	☐ No
14	Was this chest pain episode provoked by daily activities as in the case of previous chest pain?	☐ Yes	☐ No
15	Was the duration of chest pain more than 20 minutes?	☐ Yes	☐ No
16	Did the chest pain result in a pressured/crushing sensation?	☐ Yes	☐ No
17	Was the chest pain burning or stabbing?	☐ Yes	☐ No
18	Was the chest pain provoked by food ingestion or positional changes or breathing?	☐ Yes	☐ No
